# Localization of Tobacco Yellow Dwarf Virus Replication Using the In Plant Activation (INPACT) Expression Platform

**DOI:** 10.3390/v12060688

**Published:** 2020-06-26

**Authors:** Maiko Kato, Robert Harding, James Dale, Benjamin Dugdale

**Affiliations:** Centre for Agriculture and the Bioeconomy, Queensland University of Technology, Brisbane, Queensland 4000, Australia; m2.kato@qut.edu.au (M.K.); r.harding@qut.edu.au (R.H.); j.dale@qut.edu.au (J.D.)

**Keywords:** In Plant Activation (INPACT), geminivirus, replication, β-glucuronidase (GUS), tobacco, phloem

## Abstract

Geminiviruses and their diseases are a considerable economic threat to a vast number of crops worldwide. Investigating how and where these viruses replicate and accumulate in their hosts may lead to novel molecular resistance strategies. In this study, we used the Rep-inducible In Plant Activation (INPACT) expression platform, based on the genome of tobacco yellow dwarf virus (TYDV), to determine where this model mastrevirus replicates in its host tobacco. By developing an infectious clone of TYDV and optimizing its delivery by agroinfiltration, we first established an efficient artificial infection process. When delivered into transgenic tobacco plants containing a TYDV-based INPACT cassette encoding the β-glucuronidase (GUS) reporter, we showed the virus activates GUS expression. Histology revealed that reporter gene expression was limited to phloem-associated cell types suggesting TYDV replication has a restricted tissue tropism.

## 1. Introduction

Understanding virus–host interactions and the processes that occur during pathogenesis is fundamental to the development of virus control measures and ultimately resistance. Over the past twenty years, significant advances have been made in determining how circular, single-stranded (css)DNA plant viruses (including the *Geminiviridae* and *Nanoviridae* families) infect, replicate, and move throughout their hosts and the strategies they use to subvert cellular processes in their favor. Replication of the cssDNA genome is a key step in the proliferation of these viruses and occurs throughout the infection cycle, requires host cell factors, and is carried out exclusively in the nucleus [1,2]. While many studies have examined the tissue and cellular localization of different geminiviruses [3,4,5,6,7,8,9,10,11,12,13], relatively few have sought to determine whether replication of these viruses is limited to specific cell types. These latter studies have predominantly used three different approaches, in situ hybridization to detect DNA forms indicative of rolling circle replication (RCR) [14], immuno-localization based on the incorporation of the thymidine analogue, 5-bromo-2-deoxyuridine, into newly synthesized viral DNA [15], and deconstructed virus vectors capable of over-expressing a visual reporter in the presence of Rep [16,17,18].

Tobacco yellow dwarf virus (TYDV) is a dicot-infecting mastrevirus belonging to the family *Geminiviridae*. Its genome is comprised of a single component of cssDNA about 2.7 kb in size [19]. The virus encodes four proteins, a movement protein (MP), and coat protein (CP) in the virion sense and Replication (Rep) and Replication A (RepA) proteins in the complementary sense. Only Rep is essential for virus replication, however, mastrevirus RepA proteins have been shown to interact with and functionally inhibit a plant-encoded Retinoblastoma-like protein, creating a cellular environment permissive to the RCR process by which these viruses propagate [20,21]. A large intergenic region contains a stem and nonanucleotide loop structure, the origin of first strand synthesis for RCR, and transcription initiation elements for bidirectional expression of virion and complementary sense genes [20]. A small intergenic region is the site of second strand synthesis for RCR and contains features associated with termination of gene expression. TYDV causes yellow dwarf disease and summer death in tobacco (*Nicotiana tabacum* L.) and French beans (*Phaseolus vulgaris* L.), respectively [19,22,23], however, its host range is not limited to these species and has been detected in other plants such as *Raphanus* spp. and *Amaranthus* spp. [24,25,26]. TYDV is transmitted by the leafhopper *Orosius orientalis* (formerly *Orosius argentatus*), which has been reported to feed on over 60 plant species and is widely distributed in Australia, Asia, and the Pacific region [27].

While not a significant threat to global food and fiber production, its wide host range and simple genome structure has established TYDV as a useful model for geminivirus research, both as a tool to better understand viral pathogenesis and as a molecular vehicle for gene silencing [28] and manufacturing novel products in planta [29,30,31]. In the latter case, we developed a deconstructed virus vector based on the genome of TYDV termed In Plant Activation (or INPACT) as a means of biopharming a commercially important non-therapeutic protein, human vitronectin, and to express an industrial enzyme and a ribonuclease in *Nicotiana* species. The INPACT expression platform is unique in that the gene of interest is split and only reconstituted and expressed in the presence of the cognate TYDV Rep/RepA proteins. In the above cases, Rep/RepA expression was placed under the control of the alcohol-inducible *alc*A:AlcR gene switch and the proteins provided in trans within the same transgenic plant. In most transgenic tobacco lines, expression of the gene of interest was negligible in the absence of Rep/RepA but could be rapidly activated in their presence. This led us to investigate whether the INPACT system could also be utilized as a means of precisely tracking TYDV replication during an infection. In this scenario, Rep/RepA derived from the infecting virus would be responsible for activating transgene expression from the INPACT platform. Here, we describe a strategy which uses the β-glucuronidase (GUS) enzyme as a visual reporter and show Rep/RepA expressed by TYDV during an infection can activate GUS expression from an integrated INPACT platform and that replication of this virus is highly restricted to phloem-associated cell types.

## 2. Materials and Methods

### 2.1. Vector Construction

**Figure 1:**
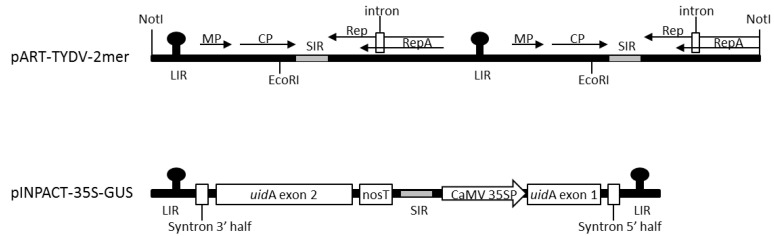
Schematic representations of the tobacco yellow dwarf virus (TYDV) infectious clone pART-TYDV-2mer and pINPACT-35S-GUS expression cassette. MP = Movement protein; CP = Coat protein; Rep = Replication protein; RepA = Replication A protein; LIR = large intergenic region; SIR = small intergenic region; Syntron = synthetic intron; nosT = Nopaline synthase terminator; CaMV 35SP = Cauliflower mosaic virus 35S promoter; *uidA* exon 1 = exon 1 of the *uid*A reporter gene encoding GUS; *uidA* exon 2 = exon 2 of the *uid*A reporter gene encoding GUS. Arrows represent coding regions. NotI and EcoRI restriction sites used to assemble the TYDV dimer clone are marked. Drawings not to scale.

Tobacco (*N. tabacum*) leaves infected with TYDV were obtained from Myrtleford, Victoria. Full-length TYDV genomic DNA was isolated from this material using the cetyltrimethylammonium bromide (CTAB) method [32] and PCR. This TYDV isolate had 95% sequence similarity to that of the TYDV GenBank accession number M81103.1 [19]. A greater-than-genome-length dimer clone of TYDV was assembled in the vector pART27. The complete TYDV genome was PCR amplified as both a NotI fragment (at position +1) using primer pairs Not-F (5′- GCGGCCGCATTAAGGCTCAAGTACCGTACGATG-3′) and Not-R (5′- GCGGCCGCATGCCTTCAGCCCCCCAGAAAACCAA-3′) and an EcoRI fragment (at position +1194) using primer pairs Eco-F (5-GAATTCTTCCACTGGTGATGTTGCTG-3′) and Eco-R (5′- GAATTCTTCCACTCTGTGCTAACCCCTA-3′). Both genomic products were initially cloned into pGEM^®^-T.Easy (Promega, Alexandria, NSW, Australia) and Sanger sequenced (Macrogen, Seoul, South Korea). The first TYDV NotI genome fragment was ligated into NotI digested binary plasmid pART27. The second TYDV EcoRI genome fragment was then ligated into the unique EcoRI site located within the first complete viral genome. The resulting dimer infectious clone was called pART-TYDV-2mer (Figure 1). Assembly of the TYDV-based INPACT vector encoding the GUS reporter enzyme, pINPACT-35S-GUS, has been previously described by [30]. A diagrammatic representation of the INPACT cassette is shown in Figure 1.

### 2.2. Transformation of Agrobacteria and Tobacco

pINPACT-35S-GUS was mobilized into *Agrobacterium tumefaciens* strain LBA4404 and pART-TYDV-2mer into *A. tumefaciens* strains GV3101, C58, and AGL1 by electroporation using the method of [33]. *N. tabacum* (cv. Samsun) was transformed using the leaf disk method of [34]. Tissue culture plants with established roots were soil acclimated and transferred to a controlled environment chamber with a 16 h photoperiod and constant temperature of 25 °C.

### 2.3. Agroinfiltration of Leaves

Tobacco leaves were agroinfiltrated essentially as described by [35]. As a mock control, leaves were infiltrated with MgCl_2_:MES:Acetosyringone (MMA) buffer alone.

### 2.4. GUS Histochemical Staining

Leaves were histochemically assayed for β-glucuronidase (GUS) expression according to [36]. Leaves were vacuum-infiltrated with a GUS substrate solution and incubated at 37 °C for 48 h then cleared in 100% ethanol for 48 h at room temperature.

### 2.5. Fixing, Sectioning and Microscopy

GUS stained leaves were cut into 5–7 mm^2^ sections and fixed in 4% formaldehyde solution for 48 h at room temperature and then cleared in 100% ethanol. Fixed leaf tissues were dehydrated through a graded ethanol series and embedded in paraffin wax. Transverse and longitudinal sections of leaves were cut using a Leica RM2245 rotary Microtome set to 5 µm. Samples were deparaffinized with xylene, and then gradually hydrated through a decreasing ethanol series (100%, 90%, and 70%) and then placed in distilled water. Leaf sections were then counter-stained in 0.5% safranin-O in 50% ethanol. After staining, slides were dehydrated using an increasing ethanol series (50%, 70%, 90%, and 100%) and then placed in xylene. Slides were mounted with dibutylphthalate polystyrene xylene (DPX) (Merck, Bayswater, Victoria, Australia), scanned with a 3D Histech slide scanner and images viewed using CaseViewer software.

### 2.6. Confirmation of TYDV Infection by PCR

Total DNA was extracted from the upper leaf of tobacco plants using the CTAB method of [37]. Approximately 1 µg of DNA was used as the template in a PCR containing GoTaq polymerase and primer pair MP-F (5′-ATGTATCCCGCCAAATACCAAGTGGTCC-3′) and MP-R (5′- TACCGGCCCGCCATTAGGGTTTCCTT-3′), which are specific to the TYDV movement protein (*mp*) gene. As a control, primer pair NtAct-F (5′-CTATTCTCCGCTTTGGACTTGGCA-3′) and NtAct-R (5′-AGGACCTCAGGACAACGGAAACG-3′) were used to amplify part of the tobacco *actin* housekeeping gene. PCR cycles were as follows: 95 °C for 30 s, followed by 30 cycles of 95 °C for 10 s, 55 °C for 30 s, 72 °C for 30 s, and a final extension of 72 °C for 2 min. PCR amplicons were electrophoresed through a 1.5% agarose gel.

## 3. Results

### 3.1. Agroinfiltration of an Infectious Clone to Establish TYDV Infection

**Figure 2:**
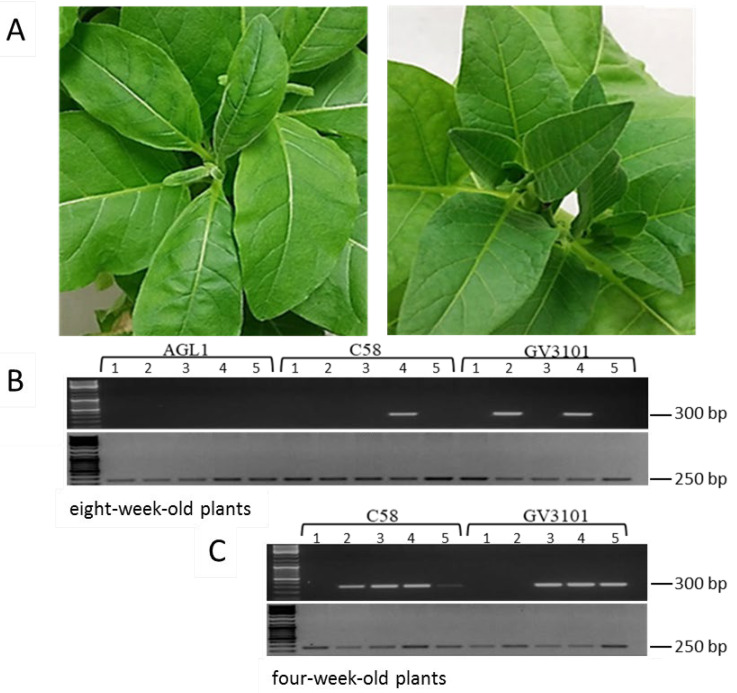
Assessment of *A. tumefaciens* strain and plant age on infection rates using agroinfiltration of the pART-2mer infectious clone in tobacco. (**A**) Leaf curling symptoms associated with TYDV infection (left) compared to the mock inoculated control (right). (**B**) PCR to confirm the presence of TYDV four weeks post agroinfiltration of the infectious clone into eight-week-old tobacco plants. (**C**) PCR to confirm the presence of TYDV four weeks post agroinfiltration of the infectious clone into four-week-old tobacco plants. Primers were designed to amplify the TYDV *mp* gene (top half of gels) and the *actin* housekeeping gene (bottom half of gels). Numbers 1 to 5 represent biological replicates.

In order to establish an efficient and reliable artificial infection system for TYDV in tobacco, three *Agrobacterium* strains (AGL1, C58, and GV3101) harboring pART-TYDV-2mer were compared for their ability to initiate virus infection via agroinfiltration. To determine whether culture density and plant age influenced infection rates, agrobacterium cultures were infiltrated at two different optical densities and plant growth points. In the first experiment, five soil-acclimatized eight-week-old tobacco (*N. tabacum* cv. Samsun) plants were infiltrated with each recombinant agrobacteria strain (optical density at a wavelength of 600 nm (OD_600_) = 0.1). Plants were monitored for symptom development and TYDV infection rates assessed by PCR at four weeks post infiltration using primers designed to amplify a 300 bp fragment of the TYDV *mp* gene (Figure 2B). To verify the integrity of the DNA, extracts were also tested with primers designed to amplify a 250 bp fragment of the *actin* housekeeping gene. In all cases, amplicons of the expected size were generated with the *actin* gene primer set. Delivery of the infectious clone in strains C58 or GV3101 resulted in extensive chlorosis in the zone of infiltration within three days of inoculation and infection rates of 20% and 40%, respectively. All plants that tested PCR positive for the virus developed distinct systemic symptoms, including stunting and curling of the new leaves, compared to the mock inoculated control line (Figure 2A). However, typical field symptoms of leaf chlorosis or yellowing symptoms were not observed in these plants. Delivery with AGL1 failed to initiate a virus infection as all five plants were PCR negative for the virus and remained symptomless. Interestingly, the leaf zones infiltrated with AGL1 showed signs of necrosis two days post infiltration perhaps representing a hypersensitive response due to incompatible host–bacteria interactions. Based on this latter finding, infiltrations with AGL1 were precluded from further experiments. In an attempt to increase the infectivity rate, five soil acclimatized four-week-old plants were infiltrated with lower densities (OD_600_ = 0.05) of agrobacteria strains C58 and GV3101 harboring pART-TYDV-2mer. Plants were monitored for symptom development and TYDV infection tested by PCR, four weeks post infiltration. Infection rates of 80% and 60% were observed in the plants infiltrated with strains C58 and GV3101, respectively (Figure 2C). Again, all plants that tested PCR positive for the virus developed typical TYDV-associated symptoms.

### 3.2. Generation of Transgenic Lines Containing the pINPACT-35S-GUS Expression Cassette

Ten independent transgenic events were generated following tobacco leaf disk transformation with pINPACT-35S-GUS and grown on Murashige and Skoog media containing the antibiotic, hygromycin. Based on their growth rates, presence of the *uid*A reporter gene (as determined by PCR), and absence of “leaky” GUS expression, four lines (lines #1, #3, #4, and #6) were selected for further analysis. The four lines were multiplied in tissue culture and acclimatized to soil for subsequent infection assays.

### 3.3. Cellular Localisation of TYDV Replication

**Figure 3:**
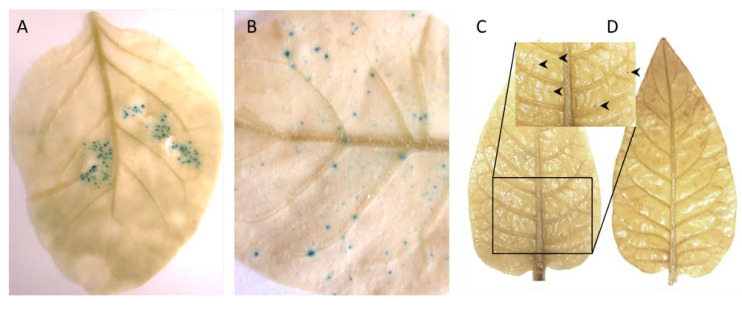
TYDV-activated GUS expression in pINPACT-35S-GUS tobacco leaves. (**A**) Activation of GUS expression in the infiltrated leaf zone five days post-delivery of the TYDV infectious clone. (**B**) GUS-expressing cells in a mature leaf following systemic movement of TYDV from the point of infiltration four weeks post-delivery. (**C**) GUS stained leaf of a transgenic pINPACT-35S-GUS plant (line #4) four weeks post-delivery of TYDV. (**D**) GUS stained leaf of a transgenic pINPACT-35S-GUS plant (line #4) four weeks post mock inoculation. Arrow heads indicate blue-stained cells. Two biological replicates of each pINPACT-35S-GUS line were inoculated with the TYDV infectious clone and one biological replicate was mock inoculated.

Four-week-old, pINPACT-35S-GUS tobacco lines #1, #3, #4, and #6 were infiltrated with *A. tumefaciens* strain C58 (OD_600_ = 0.05) harboring the infectious clone pART-TYDV-2mer. As a control, a single biological replicate of each line was mock inoculated with MMA buffer only. In one case, an inoculated leaf was detached from a transgenic pINPACT-35S-GUS plant five days post agroinoculation and GUS stained for 48 h then cleared with 100% ethanol. Intense blue-stained cells were evident only in the leaf zone agroinoculated with the TYDV infectious clone (Figure 3A). Plants were grown for approximately two to four weeks until the development of typical TYDV symptoms, including downward curving of the new leaves. No symptoms were observed in the mock challenged plants.

**Figure 4:**
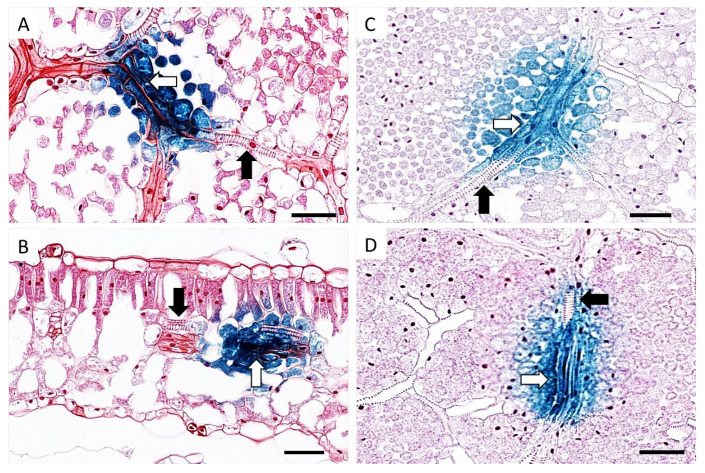
Localization of TYDV-activated GUS expression in pINPACT-35S-GUS tobacco leaves. Leaf sections showing vascular bundles consisting of xylem (black arrow) and phloem parenchyma (white arrow) cells. Longitudinal sections (**A**, **C**, and **D**) and transverse section (**B**) of GUS-expressing leaf cell zones. Scale bars = 50 µm. Two biological replicates of each INPACT-35S-GUS line were inoculated with the TYDV infectious clone and one biological replicate was mock inoculated.

At this point, the youngest unfurled leaf, a half opened young leaf and a fully mature leaf were collected from both TYDV infected and mock inoculated pINPACT-35S-GUS transgenic plants. Leaves were GUS stained for 48 h and cleared with 100% ethanol. Intense blue-stained cells were evident only in those leaves that were infected with TYDV and these GUS-expressing cells were most abundant in fully expanded mature leaves (Figure 3B and 3C). No blue-stained foci were observed in leaves from mock inoculated pINPACT-35S-GUS control plants (Figure 3D). Interestingly, these blue-stained cells were always in close proximity to the leaf veins. To visualize the GUS-expressing cells in greater detail, GUS-stained leaf tissue was fixed in 4% formaldehyde, cut into 5 µm sections, and counter-stained with 0.5% safranin-O, which stains cell walls red. Thin sections were then examined under high magnification (Figure 4A–D). Using both transverse and longitudinal sections, the blue-stained cells were confirmed to be vascular-associated and likely phloem parenchyma cells including their adjacent bundle sheath cells. No blue-stained cells were observed outside the vasculature.

## 4. Discussion

TYDV has long been considered phloem-limited based on the cytopathic effects observed in the phloem tissue of infected plants [38]. Later, this biological evidence was supported by molecular evidence which showed that expression of the TYDV Rep gene was likely restricted to vascular-associated cell types [39]. In the latter case, the authors used an autonomously replicating virus vector based on the deconstructed genome of TYDV in which the *uid*A gene, encoding the GUS reporter, was placed downstream of the promoter directing virion sense gene expression (replacing both the movement protein and coat protein gene sequences). Upon Rep expression, but in the absence of virus infection, the cassette was released from the integrated T-DNA and amplified extra-chromosomally with resultant GUS expression only in those cells undergoing episomal replication. Transgenic tobacco plants containing the cassette displayed a distinctive speckled pattern of blue staining that was often associated with leaf veins, following the addition of the GUS substrate. Here, we used the Rep-inducible INPACT expression platform to definitively prove where TYDV replication occurs during an infection. The INPACT system differs from the deconstructed virus vector strategies of [39] and those used by others [16,17,18]. In these cases, reporter gene expression was placed under the control of a subgenomic viral promoter or heterologous (CaMV 35S) promoter and basal reporter signal was amplified by Rep-mediated trans-activation and/or amplification only in infected cells. Due to the unique split gene arrangement of the INPACT cassette there is no basal reporter expression in the absence of the virus, and only those cells containing the cognate Rep display GUS activity. This feature of the INPACT system, precludes any potential misinterpretation between background and amplified reporter signal.

TYDV is naturally transmitted from plant-to-plant by the leafhopper, *Orosius orientalis*, however, rearing and maintaining these insects in a laboratory is both difficult and time-consuming. An alternative strategy to introduce a virus into a plant is by agroinfiltration, whereby *Agrobacterium* is used to deliver cloned viral DNA into the plant cell, resulting in the reconstitution of the complete virus genome and initiation of an infection. This approach has been successfully adopted for many geminiviruses; it is simple and cheap and can be used to inoculate large numbers of plants in a short period of time. However, agoinfiltration can be unpredictable and its effectiveness can vary between *Agrobacterium* strains, culture densities, plant species, type of delivery vector, age of plant, and transgene [40,41,42]. By investigating different strains of *Agrobacterium*, plant age and culture density, we developed a relatively efficient artificial infection strategy for TYDV that resulted in an 80% infection rate using four-week-old soil-acclimatized tobacco plants infiltrated with *Agrobacterium* strain C58 at a culture density of OD_600_ = 0.05.

Transgenic tobacco plants containing the TYDV-based pINPACT-35S-GUS cassette were generated and infected with the virus by agroinfiltration. Histochemical GUS staining of infected leaves showed that TYDV-derived Rep/RepA was capable of activating the integrated pINPACT-53S-GUS cassette as blue-stained cells were clearly visible throughout the leaves of infected plants only. Interestingly, these cells were always located near the leaf veins and most abundant in the fully expanded mature leaves compared to the younger leaves. This may suggest virus replication or the Rep/RepA proteins are most abundant in the older tissue types, a finding similar to that of [28], and may reflect the fact that geminiviruses alter the cellular environment of terminally differentiated cells, rather than actively expanding young leaves, to re-initiate host DNA synthesis mechanisms and their life cycles [1,15,43].

Under high magnification, GUS-expressing cells were primarily phloem-associated and not in the xylem. Transverse sections showed these cells were located in vascular bundles and not in non-phloem domains such as mesophyll and epidermal cells. This would suggest TYDV is primarily phloem-restricted, much like abutilon mosaic virus, squash leaf curl virus, and tomato yellow leaf curl Sardinia virus [6,12,16]. However, not all geminiviruses are limited to such cell types, with viruses such as tomato golden mosaic virus, beet curly top virus, maize streak virus, and bean dwarf mosaic virus able to infect a wide variety of quiescent, differentiated cell types, including mesophyll and vascular cells [4,5,7,10,11,13]. The tissue tropism of different geminiviruses is in part genetically determined by the virus itself (including both coding and non-coding viral sequences) and in some cases the developmental stage of the host [9,44].

The Rep-inducible nature of the INPACT expression platform makes it a particularly useful and versatile system for the study of cssDNA virus replication in plants. By simply replacing the origins of replication flanking the INPACT cassette, the platform could be adapted to any geminivirus or nanovirus. Further, with additional refinements, such as the use of a non-destructive reporter gene (e.g., the green fluorescent protein gene), the platform could potentially be used to track virus replication in real time throughout the plant, during the course of a natural infection. The utility of the INPACT system to be adapted into a virus-inducible resistance strategy, whereby the gene of interest encodes a toxic or anti-viral product, also remains to be further pursued from transient studies [45] to whole plants.
